# A *Drosophila* immune response against Ras-induced overgrowth

**DOI:** 10.1242/bio.20146494

**Published:** 2014-03-21

**Authors:** Thomas Hauling, Robert Krautz, Robert Markus, Anne Volkenhoff, Lucie Kucerova, Ulrich Theopold

**Affiliations:** Department of Molecular Biosciences, The Wenner-Gren Institute, Stockholm University, S-10691 Stockholm, Sweden; * Present address: Science for Life Laboratory, Karolinska Institute Science Park, S-17165 Solna, Sweden.; ‡ Present address: Institute of Neuro and Behavioural Biology, Department of Behavioural Biology, University of Münster, Badestrasse 9, D-48149 Münster, Germany.

**Keywords:** Innate immunity, Tumor, Oncogene, Insect immunity, Hemocytes, Encapsulation

## Abstract

Our goal is to characterize the innate immune response against the early stage of tumor development. For this, animal models where genetic changes in specific cells and tissues can be performed in a controlled way have become increasingly important, including the fruitfly *Drosophila melanogaster*. Many tumor mutants in *Drosophila* affect the germline and, as a consequence, also the immune system itself, making it difficult to ascribe their phenotype to a specific tissue. Only during the past decade, mutations have been induced systematically in somatic cells to study the control of tumorous growth by neighboring cells and by immune cells. Here we show that upon ectopic expression of a dominant-active form of the *Ras* oncogene (Ras^V12^), both imaginal discs and salivary glands are affected. Particularly, the glands increase in size, express metalloproteinases and display apoptotic markers. This leads to a strong cellular response, which has many hallmarks of the granuloma-like encapsulation reaction, usually mounted by the insect against larger foreign objects. RNA sequencing of the fat body reveals a characteristic humoral immune response. In addition we also identify genes that are specifically induced upon expression of Ras^V12^. As a proof-of-principle, we show that one of the induced genes (*santa-maria*), which encodes a scavenger receptor, modulates damage to the salivary glands. The list of genes we have identified provides a rich source for further functional characterization. Our hope is that this will lead to a better understanding of the earliest stage of innate immune responses against tumors with implications for mammalian immunity.

## INTRODUCTION

Most clinically manifest cancers have accumulated several genetic and epigenetic changes, which confer them a growth advantage over their neighboring cells (Hanahan and Weinberg, 2011). These changes elicit innate and adaptive immune responses both in patients and in murine and other vertebrate models (Feng et al., 2010; Hanahan and Weinberg, 2011). Both types of responses have the potential to aid in immunosurveillance, which limits cancerous growth and may even eliminate transformed cells. Conversely immune responses may contribute to tumor progression by selecting more invasive forms of transformed cells in a process called immunoediting (Schreiber et al., 2011). Human tumors and anti-tumor responses are usually studied at a late stage of tumor progression when they have accumulated several mutations and clinical symptoms have become manifest. To get access to earlier events, animal models have become increasingly important. In addition to mice these include the fruitfly *Drosophila melanogaster*, where similarities between anti-tumor and wound responses were noted and zebrafish, where neutrophil and macrophage invasion was observed at an early stage of tumor progression providing further evidence for parallels between early tumor stages and wound inflammation (Feng et al., 2010; Pastor-Pareja et al., 2008). Despite obvious physiological differences, mutations in *Drosophila* tumor suppressor genes and oncogenes can lead to tissue overgrowth and invasive behavior of transformed tissue in fly larvae and adults (Gateff, 1978; Gonzalez, 2013). Both cell-autonomous as well non-autonomous mechanisms have been shown to restrict tumor growth (Brumby and Richardson, 2003; Cordero et al., 2010; Igaki et al., 2009; reviewed by Miles et al., 2011). Our goal was to systematically study how the immune system reacts against an early stage of tumor progression, namely when the first mutations that lead to uncontrolled growth arise using *Drosophila* as a model.

Most immune responses in the fly involve a close collaboration between several immune tissues. Major immune effectors in insects comprise hemolymph-associated cells (hemocytes), the epithelial tissues, the gut and the fat body, which – analogous to the liver – secretes both inducible proteins and proteins that are constitutively secreted. Effector mechanisms include the release of antimicrobial molecules, phagocytosis, the clotting system, the encapsulation of larger objects, the formation of nodules, which sequester smaller intruders and the activation of the melanization cascade (Davis and Engström, 2012; Kounatidis and Ligoxygakis, 2012; Lemaitre and Hoffmann, 2007; Theopold et al., 2014). Encapsulation and nodulation can be considered functional equivalents of the formation of granulomas in mammals. In *Drosophila*, three classes of hemocytes, namely plasmatocytes, crystal cells and lamellocytes, participate to varying degrees in these immune reactions; all three classes of cells are involved during encapsulation (Williams, 2007). Plasmatocytes are phagocytic but also release effector molecules for example during clotting and at an early stage of capsule formation (Theopold et al., 2014; Williams, 2007). Crystal cells contain prophenoloxidase, the precursor for one of the key enzymes required during melanization and are important for capsule formation (Eleftherianos and Revenis, 2011). Finally, lamellocytes, which are rare in naive animals differentiate upon parasitization by wasps and are required to ensure complete encapsulation of the wasp eggs (Márkus et al., 2009; Williams, 2007).

Much less is known about the contribution of these effector mechanisms to the response against aberrant tissues and whether *Drosophila* uses internal cues, which indicate damage or danger to elicit immune responses. It has been suggested that the response against aberrant cells has similarities with a response against tissue damage including usage of same cues for its activation (Feng et al., 2010; Pastor-Pareja et al., 2008). Melanization as a response against aberrant tissues may occur although it is often unclear whether the reaction is induced by tumorous growth or more general changes to tissue integrity. Therefore the resulting phenotype is often described as melanotic pseudotumor (Minakhina and Steward, 2006; Watson et al., 1994). In a recent study an innate immune response against aberrant cells induced by epidermal DNA damage was analyzed. The response was shown to comprise dose-dependent melanization, an increase in hemocyte numbers and activation of JAK/STAT signaling. Subtle interactions between immunity and growth and metabolic activities were identified (Karpac et al., 2011) in line with an increasing appreciation of the complex interactions between insect immunity and physiology (Rajan and Perrimon, 2013).

Here we asked whether an immune response could be induced experimentally by expression of an oncogene in non-immune tissues. For this we expressed dominant-active Ras (Ras^V12^) in the wing discs and the salivary glands. Ras is a suitable candidate since it is mutated in a large fraction of human tumors (Miles et al., 2011). Expression of Ras^V12^ has previously been shown to induce hyperplastic growth in *Drosophila* (summarized by Miles et al., 2011). Although this is different from the highly mutated genotype of fully developed tumors, activation of Ras thus represents an early stage of tumor development. We observed the strongest effects in Ras^V12^-expressing salivary glands, where an infiltration of hemocytes takes place. Although to a varying degree, two major hallmarks of a classical encapsulation reaction are observed namely plasmatocyte spreading and lamellocyte adherence. Whole transcriptome analysis of the fat body in Ras^V12^-expressing and normal larvae confirms that a *bona fide* humoral immune response was induced. The transcriptional profile of the induced genes shows both immune signatures and unique features. Finally we provide evidence for a function of one of the induced genes in the tissue damage we observe after expression of Ras^V12^.

## RESULTS

### A *Drosophila* model for tissue overgrowth

To induce overgrowth in non-immune tissues we used a dominant active form of the *Ras* oncogene (*Ras85D^V12^*) in combination with the *Beadex*-Gal4 driver, which is expressed in wing imaginal discs and in the salivary glands where expression of Ras^V12^ leads to a developmental delay and overgrowth and inhibits autophagy (Berry and Baehrecke, 2007; see supplementary material Fig. S1 for the *Bx* expression pattern).

**Fig. 1.**
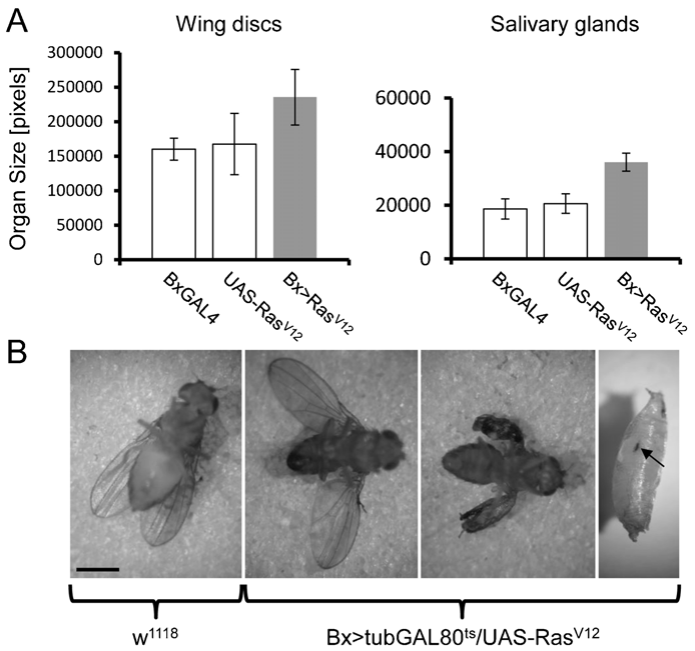
Phenotypes of Ras^V12^ transformed *Drosophila* lines. (A) Increased size of wing discs and salivary glands in Ras^V12^-expressing larvae. Wing disc and salivary gland size was determined in the indicated genotypes using ImageJ. Both Ras^V12^ wing discs and salivary glands (grey bar) are significantly larger than parental controls (white bars) (at p = 2.97 E−07 (Bx-Gal) and 0.007 (UAS-Ras^V12^), respectively, for the wing discs and 2.53 E−18 and 1.14 E−17, respectively, for the salivary glands). (B) Melanization in flies and larvae expressing Ras^V12^. Left: wild-type fly (w^1118^). Right: Bx-GAL4;tub-GAL80^ts^/UAS-Ras^V12^ flies at 18°C, 23°C and pupa at 29°C (note the melanotic spot in the pupa indicated by an arrow). Scale bar: 1 mm.

In line with previous results (Brumby and Richardson, 2003; Karim and Rubin, 1998; Pagliarini and Xu, 2003) Ras^V12^ overexpression led to an increase in the size of both wing imaginal discs but also the salivary glands in larvae (Fig. 1A) and to pupal lethality. Dead pupae also showed signs of melanization focused around two areas in the dorsal part. By inhibiting GAL4 activity with GAL80^ts^ pupal lethality was rescued in a temperature-dependent manner. Larvae raised at 18°C pupated and eclosed with wild-type wings while crosses shifted to 25°C gave rise to flies with strongly melanized wings (Fig. 1B, second right). Raising crosses at 29°C where the Gal80 inhibitor is inactive reestablished pupal lethality as observed for the cross without GAL80^ts^. This includes the appearance of two melanotic spots in the anterior part (Fig. 1B, right part).

### Ras^V12^-expressing salivary glands express metalloproteinases and damage-associated signals

**Fig. 2.**
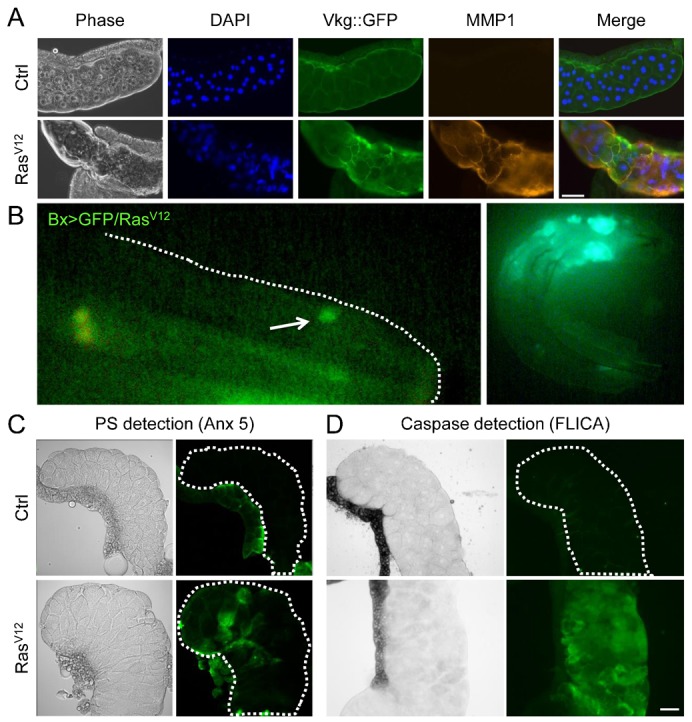
Salivary glands from *Ras^V12^*-expressing larvae produce MMP1, release tissue fragments into the hemolymph and express apoptotic markers. Salivary glands (A) from control crosses (Bx-GAL4;+/+: Ctrl) and Ras-expressing larvae (Bx>Ras, labeled Ras^V12^ in the figure) are shown in phase contrast, after nuclear staining (DAPI), in the green channel showing expression of a Viking–GFP fusion protein (Vkg::GFP) and after staining with an MMP1-specific antibody. (B) Tumor tissue in the hemolymph of *Ras^V12^*-expressing larvae. GFP-positive fragments (such as the one indicated by the arrow) are found in the hemolymph of Bx-GAL4;UAS-GFP.nls/UAS-Ras^V12^ larvae. The border of the larva is outlined with a dashed line. The figure to the right shows the whole larva. (C) Phosphatidylserine (PS) is detected with fluorophor-conjugated Annexin 5 (left) on Ras^V12^-expressing glands (Bx-GAL4>UAS-*Ras^V12^*) but not in control glands (Bx-GAL4;+/+, the borders of the glands are outlined with a dashed line, note the fat body is positive in both cases). (D) Active caspases stained with FLICA are detected in a Ras^V12^ salivary gland but not in the control. Bright field and epifluorescence image are shown in all cases. Scale bars: 100 µm.

Similar to other *Drosophila* tumor models we found that *Ras*-expressing glands produce matrix metalloproteinases (matrix metalloproteinase 1: MMP1; Fig. 2A), while imaginal discs from the same animals showed very little if any signs of expressing MMP1 (supplementary material Fig. S2A). Upon tracing with a Collagen IV–GFP fusion protein (Vkg::GFP), the integrity of the basement membrane in Ras^V12^-expressing salivary glands was more severely affected than in either discs from the same animals or control organs from normal larvae. In Ras-expressing discs, the basement membrane still formed a continuous structure, while in the glands rupture of the basement membrane occurred (Fig. 2A; supplementary material Fig. S2A; Vkg::GFP). Consistent with a disruption of the basement membrane and the MMP1-activation in Bx-GAL4>UAS-Ras^V12^-larvae, GFP-positive tissue fragments are observed in the hemolymph, when GFP is co-expressed with Ras^V12^ (Fig. 2B). Dissemination of Ras^V12^-expressing fragments has previously been shown to depend on the expression of MMP1 and is regulated through the JNK pathway (Bangi et al., 2012; Pallavi et al., 2012; Pastor-Pareja et al., 2008; Srivastava et al., 2007; Uhlirova and Bohmann, 2006). Altogether, Ras^V12^ expression in the salivary glands thus has the potential to induce an immune response since the tissue is accessible to immune effectors. We therefore focused our further analysis on this organ addressing particularly the question whether the glands undergo any changes that render them recognizable as foreign.

To characterize the changes upon Ras^V12^ expression in salivary glands, we tested for the presence of two apoptotic markers (phosphatidylserine exposure and caspase activity) both of which were detected in Ras^V12^-expressing glands and not in control glands (Fig. 2C,D). In addition nuclear fragmentation, another hallmark of apoptosis was observed in Ras^V12^-expressing glands (Fig. 2A, DAPI staining; supplementary material Fig. S3). Both the detection of caspase activity and nuclear fragmentation confirm earlier results obtained after *forkhead*-dependent Ras^V12^ expression in salivary glands (Berry and Baehrecke, 2007). Taken together this means that Ras^V12^ salivary glands express at least one damage-associated marker namely phosphatidylserine, which has the potential to elicit an immune response (Frey and Gaipl, 2011; Tung et al., 2013). We will in the following refer to the phenotype observed in these glands as “Ras^V12^-induced overgrowth”.

### The cellular response against Ras^V12^-expressing salivary glands

**Fig. 3.**
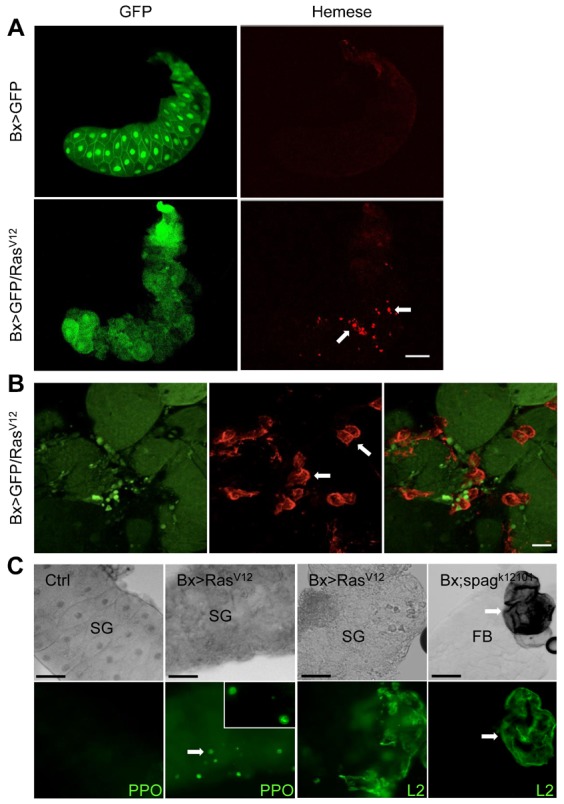
Ras^V12^-expressing salivary glands are infiltrated by hemocytes. (A) Overview of glands from *Ras^V12^* and control larvae. GFP-expressing control glands and Ras^V12^-expressing glands (the left part shows the GFP signal) labeled with a hemocyte-specific antibody (anti-Hemese, right panel, some hemocytes are indicated by arrows). (B) Hemocytes (arrows) spread around *Ras^V12^* gland cells. A detailed view of a Ras^V12^-expressing gland such as in panel A is shown. (C) Crystal cells and lamellocytes attach to Ras^V12^-expressing glands. Left part: glands (SG) from a control cross (Bx-GAL4;+/+) and Ras^V12^-expressing glands were labeled with a prophenoloxidase-specific antibody and visualized using epifluorescence. Right part: lamellocytes (L2) were visualized using a specific antibody (Kurucz et al., 2007) in Ras^V12^-expressing glands (left) and the fat body (FB) of an autoimmune mutant, which was used as a positive control (*spaghetti*, *spag^k12101^*). Scale bars: 100 µm (A), 20 µm (B), 50 µm (C).

Since MMP expression has been shown to correlate with hemocyte recruitment to aberrant tissues in somatic recombination models (Pastor-Pareja et al., 2008), we asked whether the same occurs in Ras^V12^-expressing salivary glands. For hemocyte detection we used a Hemese-specific antibody (Kurucz et al., 2003), which has previously been found suitable to characterize melanotic pseudotumor mutants (Minakhina and Steward, 2006). Indeed, albeit to a variable extent we detected infiltration of hemocytes in all Ras^V12^-glands and not in control glands (Fig. 3A). This shows that a cellular immune reaction against overgrowth had been initiated. In contrast, despite the fact that Ras^V12^-expressing imaginal discs also contained attached hemocytes we were unable to detect any significant differences compared to control discs, which showed comparable hemocyte counts (supplementary material Fig. S2B). Hemocytes had also started to spread around Ras^V12^ salivary gland cells (Fig. 3B) reminiscent of what occurs during encapsulation of wasp eggs (Williams, 2007). In addition we found crystal cells attaching to Ras^V12^-expressing glands (Fig. 3C, left). Of note, despite the presence of crystal cells larval glands lacked any signs of melanization although this was subsequently observed in pupae (Fig. 1B). Finally, we also detected lamellocytes on both Ras^V12^ salivary glands and on the fat body of *spag* mutant larvae, which were included as a positive control for a melanotic pseudotumor (Fig. 3C, right). Despite the fact that hemocytes had been recruited to Ras^V12^-expressing glands, total hemocyte counts did not differ compared to control larvae (supplementary material Fig. S4). Taken together these results show that the cellular response against aberrant salivary glands shares several similarities with the granuloma-like encapsulation reaction.

### The humoral response against Ras^V12^-expressing cells

**Fig. 4.**
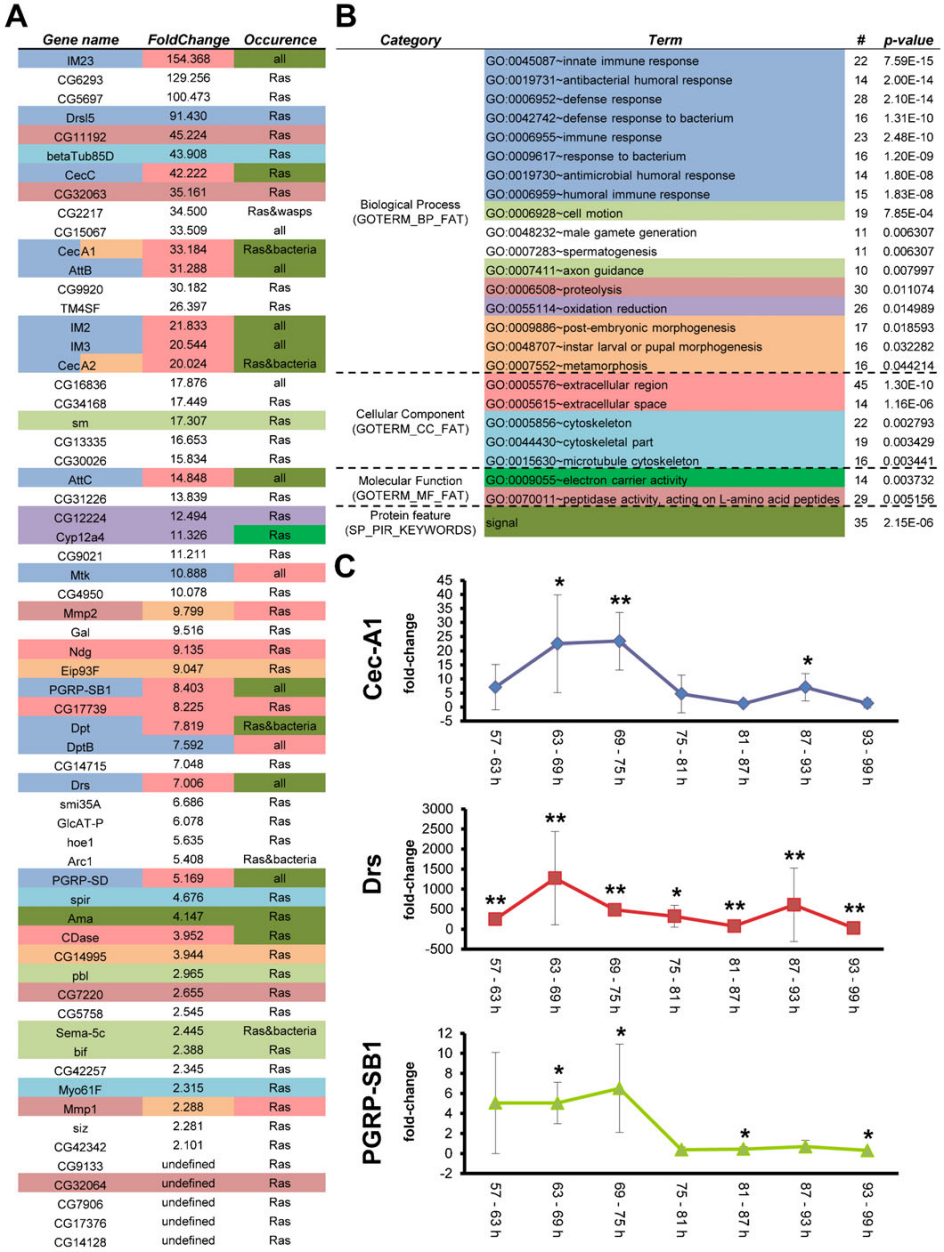
Induction of a systemic immune response in Ras^V12^-expressing larvae. (A) List of genes that are significantly induced (at q<0.05 and induction level >10) in the *Ras^V12^*-expressing glands (40 larvae were used for each replicate). (B) The fat body transcription profile of *Ras^V12^*-expressing larvae shows the hallmarks of an immune response GO classification of genes that are differentially expressed after Ras expression was performed using DAVID (see Materials and Methods for details). The most significant classifications are indicated (see supplementary material Table S2 for a more extensive list of annotations. (C) Induction kinetics of selected immune genes in Ras^V12^-expressing larvae. Individual analysis of a Toll-regulated (Drosomycin) and two preferentially imd-dependent genes (Cecropin A1 and PGRP-SB1) confirms their differential regulation and reveals a complex pattern during the course of the 3^rd^ instar. The ratio of expression between *Ras^V12^*-expressing and control larvae was determined at the indicated time points after hatching. (Significance levels are: for CecA1: 0.0831; 0.0013; 0.0403 for Drs: 0.0015; 0.0035; 2.15 E−6; 0.0374; 0.0001; 0.0097; 0.0015 and for PRGP-SB1: 0.0151; 0.0167; 0.0321; 0.0436, Student T-test; unpaired, equal variance.)

To find out whether the cellular response against Ras^V12^-induced overgrowth is accompanied by a humoral response, we examined the proteome and transcriptome of fat bodies from Ras^V12^-expressing and control larvae. At the protein level no differences in protein production were observed (supplementary material Fig. S5). For a comprehensive transcriptome analysis, fat bodies were dissected in triplicates from Ras^V12^-expressing and normal wandering third instar larvae and the expression pattern compared. Fig. 4A shows genes that differ more than twofold and at q<0.05. Altogether we identified 63 genes that were differentially regulated including many known immune genes. We also observed that the transcriptional profiles of control samples were more consistent than those of fat bodies from Ras^V12^-expressing larvae although both sets had been prepared from identical developmental time points. This is in line with the previously observed variability of the pseudotumorous phenotype (Watson et al., 1991; Watson et al., 1992). To more comprehensively cover genes that were induced in Ras-expressing animals, we also analyzed genes that differed more than two fold at p<0.05 compared to wild-type larvae. This less stringent analysis identified 438 genes (supplementary material Table S1). In both lists, the genes that were differentially regulated showed substantial overlap with previously characterized immune responses after infection with common Gram negative (G−) and Gram positive (G+) bacteria (Irving et al., 2005; Vodovar et al., 2005) and wasps (Lee et al., 2011; Schlenke et al., 2007; Wertheim et al., 2005) and contained many known immune effectors and regulators (Fig. 4A,B; supplementary material Table S1). Among these, known antimicrobial peptides (AMPs) such as Cecropins and Attacins as well as a previously described class of immune-induced peptides of unknown function (immune-induced molecules, IMs, (Uttenweiler-Joseph et al., 1998)) were highly represented (Fig. 4A; supplementary material Table S1). In addition four recognition proteins were up-regulated including two PGRPs (PGRP-SB1 and PGRP-SD; Fig. 4A) and two CD36-like scavenger receptors (Santa-maria and CG7227 (Nichols and Vogt, 2008); supplementary material Tables S1 and S2). Modular Enrichment Analysis confirmed that Ras-induced genes belonged primarily to immune-related GO categories (Fig. 4B; supplementary material Table S2). In addition to known immune genes, a significant fraction is specifically induced in the presence of Ras^V12^-expressing tissues (394, genes in the Venn diagram; supplementary material Fig. S6), including genes with a function during spermatogenesis and development, as well as genes of unknown function.

Differential expression in Ras^V12^-expressing larvae was confirmed for three immune genes using qPCR of RNA from the last larval instar. We tested two highly induced AMPs (Cecropin A1 and Drosomycin), which are regulated by both the imd and Toll pathways and a recognition molecule (PRGP-SB1), which is primarily induced via the imd pathway (De Gregorio et al., 2002). To obtain more refined insight into the kinetics of the response against transformed tissue RNA was collected at 6 hour intervals (Fig. 4C). The pattern of induction along the complete third larval instar confirms the heterogeneity we had previously observed (see above) indicating that the sequencing data covered different time points from the kinetics. The most significant differences in the kinetics study were observed between 69–75 hours after egg-laying. Both AMPs (CecA1 and Drs) as well as PGRP-SB1 were induced at this time. The AMPs showed a second increase in expression between 87–93 hours indicating that the response might comprise more than one phase. Taken the genome-wide transcriptome study and the qPCR results together, we conclude that both imd- and Toll-dependent genes are induced. To test the possibility of external infections as a source of immune-induction, we included GFP-expressing entomopathogenic bacteria (*Photorhabdus luminescens*) when raising Ras^V12^-expressing larvae. We observed neither an increase in mortality nor any signs of septicemia indicating that the bacteria had not gained access to the hemolymph. Instead, GFP-expressing *Photorhabdus* appeared to be cleared from the gut within 24 hours (supplementary material Fig. S7). To address whether small immune elicitors could pass the salivary glands into the hemolymph we employed an assay established for adult flies (Rera et al., 2012). Larvae were fed on Brilliant blue FCF supplemented fly food, which can be followed in vivo and found Ras^V12^-glands to be free of leakages (supplementary material Fig. S8). Finally we grew Ras^V12^-expressing larvae on food containing antibiotics and found that the induction levels of Drs as a proxy for Toll-activation did not differ from untreated samples although bacteria had been successfully eliminated (supplementary material Fig. S9). Even the melanotic spots in Ras-expressing larvae were still visible after depletion of bacteria. Taken together this shows that in all likelihood the transcriptional profile reflects a genuine response towards the transformed tissue. It appears that at the humoral level the response against Ras^V12^-induced overgrowth bears hallmarks of both an immune response and responses that are involved in developmental processes. Manual inspection of the genes of unknown function that were induced in Ras^V12^-expressing larvae, showed that a large fraction of them are induced during normal development in the fat body of either white prepupae, late pupae or both. Several immune genes (Dro5, Cecropins A1, A2 and C and PGRP-SD) show a similar increase in constitutive expression during pupal stages in wild-type animals. Altogether the fat body response in Ras^V12^-expressing larvae shows a highly significant enrichment for immune-related genes. In addition, a large number of genes are specifically induced in response to the Ras-expressing tissues providing a rich source for further functional characterization.

### The phenotype of Ras^V12^-expressing glands is modified in *santa-maria* mutant backgrounds

**Fig. 5.**
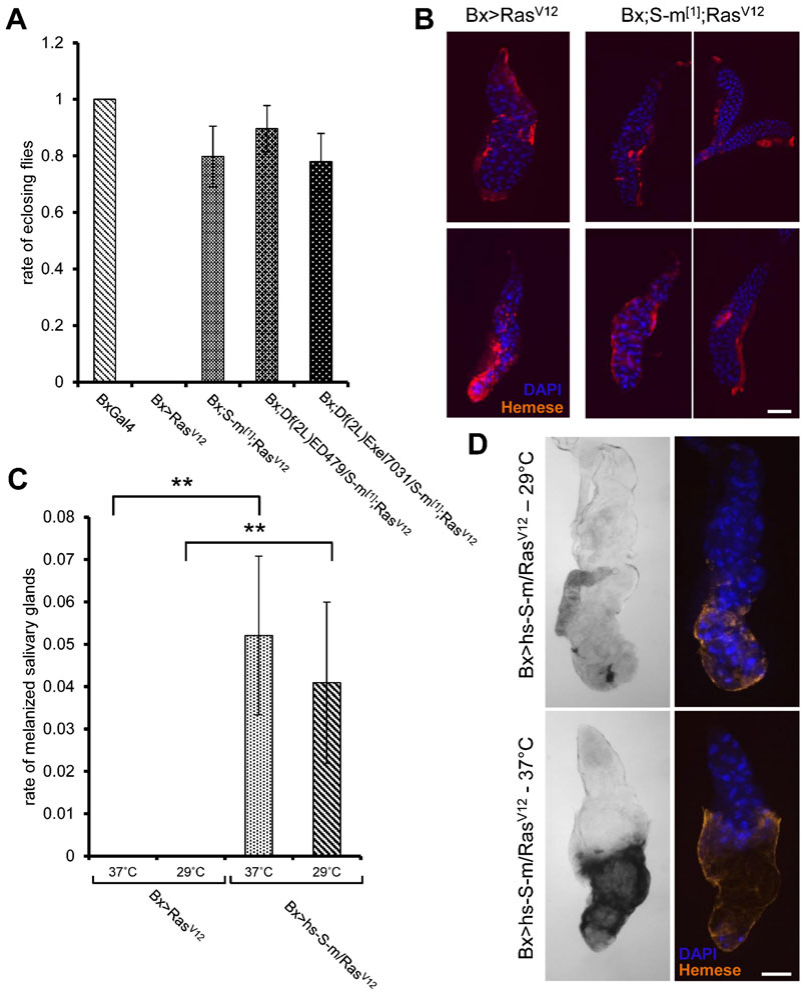
Santa-maria modifies the phenotype of Ras^V12^-expressing larvae. (A) Increased survival rate of Ras^V12^-expressing larvae in a *santa-maria* mutant background. The percentage of eclosed flies at 25°C is shown for the indicated genotypes. (B) Partial restoration of normal histology of *Ras^V12^* salivary glands in a *santa-maria* mutant background. The hemocytes are labeled with the Hemese antibody and nuclei are stained with DAPI. Ras^V12^-expressing glands were combined with the mutant background at either 25°C or 29°C (note the more regular pattern of DAPI staining in the mutant background, which was observed in about 1/3^rd^ of the glands, shown in the right part but not in others shown in the left part). (C) Overexpression of Santa-maria leads to melanization of Ras^V12^-expressing salivary glands at the larval stage. The rate of melanized glands is shown for the indicated genotypes. The difference between Ras^V12^-expressing larvae before and after overexpression of Santa-maria is 0.005 and 0.008 for 37°C and 29°C, respectively. (D) Induction of melanization in Ras^V12^-expressing larvae after induction of Santa-maria using heat shock (BxGal4/+;;hs-santa-maria/UAS-Ras85D^V12^) at 29°C or after heat shock (37°C), representative melanized salivary glands are shown in transmission light and after staining with anti-Hemese to reveal plasmatocytes and DAPI for nuclear staining. Scale bars: 200 µm.

As a test for functional importance in our *Ras* model, we decided to study one of the genes, which lacked strong support for an immune function. The scavenger receptors of the CD36 class appeared to be likely candidates for regulating the response, in particular, because we had observed that phosphatidylserine, which is a common ligand for CD36 members, is expressed in Ras^V12^ glands. Viable mutants in the CD36 member *santa-maria* were combined with Ras^V12^-expression asking whether this modified the phenotype of Ras^V12^-expressing larvae. In addition Santa-maria had been found induced after wasp infestation, a response that according to the data shown above appeared akin to the response against Ras-expressing glands. Supporting a positive regulatory function for Santa-maria during Ras^V12^-induced overgrowth, *santa-maria* homozygous larvae showed a partial rescue of the phenotype after Ras^V12^-expression, including the aberrant histology of the glands and the mortality (Fig. 5). Conversely larvae where Santa-maria was overexpressed using heat-shock drivers along with expression of Ras^V12^ at 29°C, showed melanization of salivary glands in about 4% of cases in contrast to larvae that expressed Ras^V12^ alone at the same temperature and which did not show any signs of melanization (Fig. 5C). The fraction of melanized glands was slightly although not significantly increased after heat-shock at 37°C. In contrast to its frequency, melanization was more extensive at 37°C (representative samples are shown in Fig. 5D). Taken together this indicates that *santa-maria* acts as a modifier of Ras^V12^-induced tissue abnormalities in different genetic backgrounds (overexpression versus homozygous and transheterozygous lines) and that this affects the survival of mutant larvae. Altogether this provides general proof-of-principle that the genes we identified as part of the response towards aberrant tissues can be studied at the functional level and that Santa-maria in particular acts as a positive regulator of the phenotypes we observe upon Ras^V12^-expression.

## DISCUSSION

The aim of this work was to experimentally induce an early stage of tumor development in vivo and study the ensuing immune response. For this, we expressed a dominant-active form of *Ras^V12^* in imaginal discs and salivary glands. Against our initial expectation, although imaginal disc cells are proliferative and therefore expected to be more susceptible to expression of an oncogene than polytenic salivary gland cells, we observed the strongest effects in the glands, which grew larger, expressed apoptotic markers and metalloproteinases. Similar to our findings, induction of metalloproteinases and dissemination of hindgut cells has also been observed after expression of Ras^V12^, which synergizes with an inflammation-like response (Bangi et al., 2012). In our hands, the changes at the cellular level correlated with the strength of the cellular response, which was directed mostly against the glands. Despite the lack of a response towards larval wing discs some effects were observed on wings in adult flies, which displayed melanotic spots. We hypothesize that due to the lack of metalloproteinases (Fig. 2; supplementary material Fig. S2), any changes in transformed imaginal disc cells are not accessible to the immune system. In contrast, during disc eversion in the pupae, expression of metalloproteinases and the resulting degradation of the basement membrane (Pastor-Pareja et al., 2004; Srivastava et al., 2007) may render the same cells accessible to effector mechanisms such as melanization. This is different in Ras^V12^-expressing salivary glands, which overgrow, express apoptotic markers and metalloproteinases already during the last larval instar. In addition by expressing phosphatidylserine (Fig. 3C), the glands have the potential to induce melanization (Bidla et al., 2009). Supporting a dual requirement for (i) damage-associated patterns such as phosphatidylserine and (ii) accessibility of those signals for immune cells, we could not observe any signs of melanization when apoptosis was induced by overexpression of either Grim or Hid with the Beadex driver or disc-specific drivers (supplementary material Table S3). Only hemocyte-specific expression or heat shock-activation of apoptotic inducers, which also affects hemocytes led to melanization, in line with our own previous observations (Bidla et al., 2007; Bidla et al., 2009). Taken together, the expression of Ras^V12^ leads to changes in the salivary glands, which are shared with tumor cells and have the potential to induce an immune response. These changes may be due to the early onset of Beadex expression during development (Graveley et al., 2011) or to the fact that expression of Ras leads to continued growth of salivary gland cells at a stage when they normally activate autophagy as shown before (Berry and Baehrecke, 2007). Supporting a role for the early onset of Beadex expression, we did not observe similar changes in salivary glands when using a strong driver that is regulated by ecdysone and expressed only at the last larval stage (salivary gland secretion 3, SGS3).

We observe a robust cellular response towards overgrowing salivary glands that express Ras^V12^ and are regulated by Bx. At the cellular level, this response shows similarities to an encapsulation of foreign tissues such as wasp eggs: (1) initially plasmatocytes are attracted, which attach to the aberrant tissue and spread around it (Fig. 3); (2) the presence of overgrowing cells induces the differentiation of lamellocytes and recruitment of crystal cells, which attach to the glands (Fig. 3C). The transcriptional profile of induced genes in the fat body of Ras^V12^-expressing larvae also shares some similarities with the pattern found upon wasp parasitization although many bacterially-induced genes were also identified as well as genes involved in developmental processes and potentially in wound healing. In contrast to many mutants that lead to melanotic pseudotumors where it was difficult to decide whether melanization is due to changes in the affected tissue or in immune cells themselves, we are able to ascribe the response we observe to changes in the target (non-immune) tissue. In fact, even hemocyte numbers do not appear to differ between Ras^V12^-expressing and control larvae eliminating hemocyte proliferation as a cause for the reaction we observe against the glands (supplementary material Fig. S4). Taken together this proves that the modification of cells by expression of an oncogene and the immune response against them can be physically separated in fly larvae and establishes a useful model to study this response.

The response in the fat body of Ras^V12^-expressing larvae shows typical signatures of an immune response and is dominated by the expression of peptides with known antimicrobial activity such as Cecropins and Attacin and other previously described immune-induced peptides (Uttenweiler-Joseph et al., 1998). We observe strong activation of both the Toll and imd pathways. Induction of Toll-dependent expression is in line with several previous reports on a correlation between activation of melanization and Toll-signaling (Govind, 1996; Lemaitre et al., 1995; Scherfer et al., 2006).

It remains to be studied which of the induced genes are active against the Ras-expressing tissue, but one possibility is that some of the peptides act against aberrant cells as has been hypothesized (Reddy et al., 2004). For example due to their basic nature antimicrobial peptides may have the potential to specifically bind to phosphatidylserine-positive tumor cells (Zwaal et al., 2005). As mentioned above, we observe that phosphatidylserine is exposed in Ras^V12^-expressing glands similar to other pathological situations (Zwaal et al., 2005) making them a potential target in this scenario. In a similar way CD36-like scavenger receptors, which we found differentially expressed in Ras^V12^-expressing larvae may interact with phosphatidylserine. Using a refined kinetics to study transcription we observe some variability indicating that antimicrobial peptides are not expressed to the same extent during all stages of the response (note the biphasic expression pattern in Fig. 4). Interestingly, we noticed that the majority of the genes with “unknown function” that are induced in the presence of Ras^V12^-expressing glands are also up-regulated in the fat body of wild-type pupae during normal development (Graveley et al., 2011; FlyBase). This is compatible with the idea that some genes required for tissue reconstruction during metamorphosis are also recruited for the response against Ras^V12^-induced cellular changes.

Both these genes and the immune genes are expected to include interesting candidates for regulators and effectors of the innate response against aberrant cell growth. As a proof-of-principle, we show that a mutation in one of them (*santa-maria*) modulates the cellular phenotype after Ras^V12^-overexpression and reduces lethality. Thus Santa-maria contributes to the aberrant phenotype in Ras^V12^-expressing salivary gland cells although it remains to be determined how this works at the molecular level. Based on previous work (Halme et al., 2010), we propose a model where Santa-maria modulates the synthesis of retinoic acid. In this scenario, ectopic expression of Ras^V12^ using Bx activates retinoids similar to a wound model that involves Bx-dependent expression of apoptotic inducers, which leads to a delay in pupariation (Halme et al., 2010; supplementary material Table S3). In contrast to apoptotic wounds, though, Ras^V12^-expressing glands fail to heal leading to pupal lethality. This is in line with the classical proposal that tumors can be regarded as wounds that do not heal (Dvorak, 1986).

### Conclusions

**Fig. 6.**
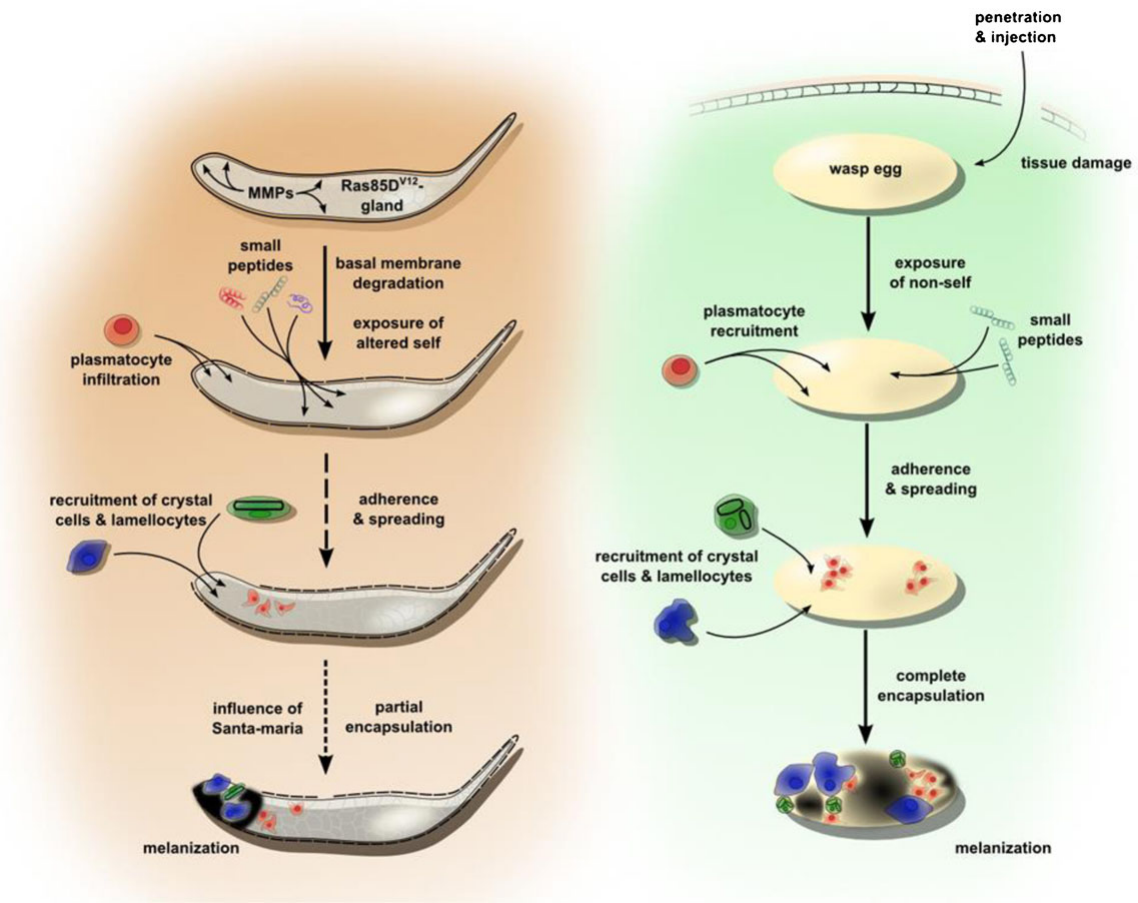
Comparison between the response against transformed tissues and capsule formation. At the cellular level the two responses share many similarities including the cell types involved and the kinetics of their recruitment. The encapsulation of transformed tissue is generally less extensive and melanization less frequent. Specifically in the presence of transformed glands, we observe induction of a large set of small peptides, several immune proteins (such as the scavenger receptors) and a large fraction of proteins of unknown function.

Altogether this work provides further evidence that *Drosophila* is a useful model to study the immune response against aberrant cell growth such as Ras^V12^-induced cellular changes, in particular during early stages, which are less amenable in mammalian models. By characterizing both the cellular and the humoral branch, we are able to establish a comprehensive view of this early response against Ras-expressing cells, which appears to involve both genes required during other immune responses as well as genes of hitherto unknown function in immunity. Primarily at the cellular level we find similarities to the encapsulation response, which helps to segregate and inactivate large intruders such as wasp eggs within the insect host and includes melanization (Fig. 6). Melanization is also the ultimate stage of the response described here and earlier stages likely involve some of the induced peptides as well as proteins of unknown function (Fig. 6). Possibly, the earliest phase of the response involves scavenging transformed cells. Studying *Drosophila* larvae bearing mutations in the genes we identified and their effects on the transformed phenotype will provide further mechanistic insight into the response against cell damage and possibly anti-tumor responses in invertebrates with clear implications for vertebrates and identify novel targets to modulate these responses.

## MATERIALS AND METHODS

### *Drosophila* stocks

Bx^MS1096^-GAL4, LzGal4,UAS-mCD8::GFP.L, UAS-Ras^V12^, spag^k12101^/CyO,UAS-GFP.nls, santa-maria^1^, and the deficiency lines Df(2L)Exel7031/CyO and Df(2L)ED479, P(3′.RS5+3.3′) ED479/SM6a, which cover the santa-maria locus, were obtained from the Bloomington Stock Center (USA). UAS-grim was obtained from the Vanderbilt University Medical Center, Nashville, Tennessee 37232). The vkg^G00454^-GFP-trap line was obtained from from FlyTrap (Quiñones-Coello et al., 2007). The hs-santa-maria strain was a kind gift of Craig Montell (Wang et al., 2007). Generated strains include: Bx-GAL4;Bc^1^, Bx-GAL4;tub GAL80^ts^, Bx-Gal4;santa-maria^1^,Bx-Gal4;;hs-santa-maria, santa-maria^1^;UAS-Ras^V12^, Bx-Gal4;Df(2L)ED479/CyO,GFP and Bx-Gal4;Df(2L)Exel7031/CyO,GFP. All stocks were maintained on a standard potatomash/molasses medium at 25°C.

### Staining and imaging

The following primary antibodies and dyes were used: mouse monoclonal anti-Hemese (1:5 dilution (Kurucz et al., 2003)); mouse monoclonal anti-L2 (1:50 dilution (Kurucz et al., 2007)); mouse monoclonal anti-PPO (1:200 dilution, gift from M. Crozatier); mouse monoclonal anti-Mmp1 (1:50 dilution, Developmental Studies Hybridoma Bank, University of Iowa developed by Andrea Page-McCaw); rabbit Cy3-conjugated anti-mouse IgG (1:200 dilution, Jackson Immuno Research); rabbit FITC-conjugated anti-mouse IgG (1:200 dilution, Jackson Immuno Research and Sigma); AlexaFluor 546-conjugated anti-mouse (1:800 dilution, Molecular Probes) DAPI (1:5000 dilution, Sigma–Aldrich), Hoechst 33342 (1:1000 dilution, Immunochemistry); FLICA reagent (1:150 dilution, Immunocytochemistry); AlexaFluor-conjugated Annexin-5 (1:250 dilution, Invitrogen). The samples were mounted in DABCO-glycerol or in Fluoromount-G (SouthernBiotech). Bright field, phase contrast and wide field fluorescence images were taken in a Zeiss Axioplan 2 microscope equipped with a motorized stage and AxioVision™ software package installed (version 4.6.3 including modules for Z-stack recording and extended focus). Confocal images were taken in a Zeiss LSM 510 Meta microscope.

### Live-imaging of larvae

Larvae were fixed on Scotch tape that was glued onto microscope slides. Live *Bx*>*GFP* and *Bx*>*GFP Ras^V12^* larvae were imaged with a 2.5× objective in a Zeiss Axioplan 2 microscope and an AxioCam HRm camera.

### Detection of hemocytes and MMP1 on tissue

Salivary glands and wing discs were dissected in a depression slide with larvae immersed in PBS. Tissues were fixed in 3.7% formaldehyde 1 h at RT or overnight at 4°C, washed three times 10 min in PBS and blocked in PBS, pH 7.5 containing 1% w/v BSA for 1 h. This step was followed by incubation with primary antibody (diluted in PBS, pH 7.5 containing 1% w/v BSA) at 4°C, overnight. Secondary antibody (diluted in PBS, pH 7.5 containing 1% w/v BSA) was added for 1 h. Nuclei were stained with Hoechst 33342 or DAPI. Following three times 10 min washing samples were mounted in DABCO-glycerol or in Fluoromount-G (SouthernBiotech).

### Determination of tissue size

Bright field images of whole glands mounted with a coverslip were taken in a Zeiss Axioplan 2 microscope using a Neofluar 2.5× objective and an AxioCam HRm camera. The total area per gland was measured in pixels by masking each gland using the “freehand tool” in Image J (http://rsbweb.nih.gov/ij). The average gland size per sample was determined by measuring at least 13 salivary glands.

### Quantification of hemocyte adherence on wing discs and salivary glands

Hemocytes and nuclei were labeled and mounted as described above. Images of whole glands were generated by recording image stacks of 1.8 µm and extended-focus images were calculated (wavelets method, no alignment). Hemocytes on glands were marked and their cumulative area measured in square pixels by ImageJ. The mean of the surfaces covered by hemocytes of all glands measured was divided by the average gland size. Since hemocyte numbers in *Drosophila* larvae are not normally distributed but follow a log-normal distribution (Sorrentino, 2010), all area values were log-transformed. These values were plotted and standard deviation was calculated based on these values. At least 11 salivary glands were analyzed per group.

### FLICA method

Salivary glands were washed in PBS once. FLICA™ reagent (SR-FLICA, Poly-Caspase Kit, cat. no. 916, *Immunochemistry*, *pan-caspase assay*) was diluted 1:150 in PBS and added to the samples and incubated for 60 min at room temperature to allow for reagent binding to active caspases. Samples were washed in 1× washing buffer, and fixed with a formaldehyde-based fixative. Both washing buffer and fixative were supplied with the kit. Hoechst 33342 was used to stain nuclei. Annexin V method: Annexin V binding buffer from the ApoAlert^Tm^ kit was used (Clontech) in conjunction with Alexa Fluor conjugated Annexin V (Invitrogen). Salivary glands were incubated in Annexin V binding buffer solution (2.5 µL Annexin V/100 µL binding buffer) for 15 min at room temperature and protected from light. Salivary glands were washed in binding buffer and fixed in 2% formaldehyde, then washed two times. Nuclei/DNA was stained with Hoechst 33342, samples were washed and mounted in Fluoromount-G (SouthernBiotech).

### Hemocyte counts

Wandering stage third instar larvae were collected as described above washed with fresh water and then they were bled into 20 µl PBS by ripping the cuticle with forceps. The solution was transferred to a counting chamber (FastRead 102 counting slides; Immune Systems Ltd.) and hemocytes were counted in all grids with a phase-contrast microscope. The number of lamellocytes was recorded during all hemocyte countings using a phase-contrast microscope; all cells that had elongated and large-flattened morphology were considered as lamellocytes. The hemocyte numbers were calculated based on to the manufacturer's instructions, taking into account that on average 0.3 µl of hemolymph is retrieved from one larva. To reduce the variance, the hemocyte counts were log-transformed for statistical analysis (Sorrentino, 2010; Fig. 6). ANOVA was performed on the log-transformed data (Sigmaplot 12). Additionally a Kruskal-Wallis One Way Analysis of Variance on Rank test was done with the raw hemocyte numbers to confirm the results (Sigmaplot 12).

### RNA extraction, RNA sequencing and bioinformatics analysis

Fat body tissues, excluding gonads, were collected in triplicates immediately after dissection in ice chilled TRIzol® Reagent (Invitrogen, cat. no. 10296-028). After collecting fat body from 40 individuals for each replicate, samples were vortexed and stored at −80°C. After thawing the total RNA was prepared according to the instructor's manual for TRIzol® Reagent and further purified with the help of the Qiagen RNeasy Mini Kit (cat. no. 74104). Qiagen DNase for DNA degradation and subsequent purification was applied. cDNA was prepared employing the SuperScript III Reverse Transcriptase (Invitrogen, cat. no. 18080-044). Quality was assessed using the Experion RNA StdSens Analysis kit (BioRad).

### Sequencing

The clustering was performed on a cBot cluster generation system using a HiSeq paired-end read cluster generation kit according to the manufacturer's instructions. The samples were sequenced on an Illumina HiSeq 2000 as paired end reads to 100 bp. All lanes were spiked with 1% phiX control library. The sequencing runs were performed according to the manufacturer's instructions.

### Bioinformatics analysis

Base conversion was done using Illuminas OLB v1.9. Mapping of the raw reads obtained from the Illumina HiSeq 2000 sequencing system was performed by Tophat v1.0.14 (Trapnell et al., 2009). Bam files were generated where reads were mapped to the chromosome and duplicates were removed. To obtain quantification scores for all *Drosophila* genes, FPKM (fragments per kilobase of exon model per million mapped reads) values were calculated using Cufflinks v0.0.5 (Trapnell et al., 2012). Ensembl build *EMBL Drosophila_melanogaster.BDGP5.25.64.gtf* was used as a reference genome. Differential gene expression analysis was performed using Cufflinks, Cuffcompare and Cuffdiff (Trapnell et al., 2012), setting the false discovery rate threshold for significance at q = 0.05, the minimum alignment count to 10 and including quartile normalization (http://rsbweb.nih.gov/ij). The fold change in gene expression was calculated by dividing the FPKM values. This value was used to sort and separate up-regulated from down-regulated genes. Lists of significantly up-regulated genes were subjected to Modular Enrichment Analysis via David. The sequencing data (fastq files), the gene expression pattern of all samples (output file of Cufflinks “gene expression” converted to Excel) and the results (output file of Cuffcompare “gene differential expression testing” converted to Excel) are uploaded to the NCBI, GEO under the accession number GSE4148.

### Real time qPCR

RNA extraction, purification, cDNA synthesis and assessment were carried out as described above for 12 larvae per time frame and genotype. Real time qPCR was performed, comprising KAPA PROBE FAST Universal qPCR Master mix and the TaqMan Gene Expression Assays for the indicated genes, namely PGRP-SB1 (Dm01805870_g1), or customized probes and the corresponding primers for CecA1 and Drs on a Rotor-Gene Q (Qiagen). All samples were analyzed in triplicates and normalized to the expression of RpL32 as an internal control. Microsoft Excel served to analyze the obtained data.

### Survival rate

For survival rate assays, pupae and eclosing flies were counted and the ratios relative to the total number of individuals determined.

### Heat shock treatment

Eggs were collected on plates during 6 h and hatching larvae carefully transferred to new vials to avoid larval crowding. Heat-shocks were applied according to Gong and Golic at 72 h and 96 h after egg deposition (Gong and Golic, 2006). Incubation of vials for 60 min in a 37°C water bath was preceded by administering 35°C for 30 min in a separate bath to minimize heat induced mortality. Control larvae were incubated at 29°C like the experimental samples before and after heat treatment.

### SMURF-assay

Eggs of the appropriate genotypes were collected for 6 h at 25°C and raised at 29°C on standard potatomash/molasses medium, while carefully avoiding larval crowding. Exactly 96 h after egg deposition larvae were transferred to standard medium supplemented with Brilliant blue FCF (25 mg·ml^−1^) (Rera et al., 2012) (Erioglaucine disodium salt, 861146, Sigma–Aldrich, Co.). After 1, 3 or 24 h at 29°C on this blue medium, the individual larvae were washed thoroughly in water and 75% ethanol to remove any dye on the cuticular surface. All specimens were anaesthetized for maximally 3 min in special cages with diethylether before taking pictures of the whole organism with a Leica MZ FLIII equipped with a LumixG2. LzGal4,UAS-mCD8::GFP-larvae were used because of their strong GFP-expression in salivary glands, which facilitates detection of injuries. Glands were fragmented by in situ, non-invasive wounding (squeeze wounds) without opening the cuticle right before transferring to Brilliant blue FCF-supplemented medium.

### Raising larvae on antibiotics

Eggs collected during 6 h time slots were incubated for 24 h at 25°C and hatched larvae transferred to antibiotics medium, while carefully avoiding larval crowding. Antibiotics added comprise Neomycin (50 mg·ml^−1^), Vancomycin (50 mg·ml^−1^), Carbenicillin (50 mg·ml^−1^) and Metronidazole (8.38 mg·ml^−1^) (Ryu et al., 2008). To check Drosomycin expression, larvae were incubated for another 48 h at 29°C and treated as mentioned above. For the plating assay, larvae were raised for an additional 72 h at 29°C. At this time larvae were washed by several times mild vortexing in 75% ethanol to remove bacteria and fungi attached to the cuticle. Individual larvae were subsequently homogenized in 100 µl PBS and the homogenate plated under sterile conditions. After formation of bacterial colonies cfu-counts were analyzed with ImageJ. Unpaired Students T-tests with equal variance were performed for comparison between the different samples. Additional batches of 72 h antibiotics treated larvae (29°C) were shock frozen in liquid nitrogen. Genomic DNA of these samples was extracted with Phenol-chloroform-isoamyl alcohol [77617, Sigma–Aldrich, Co.] and used for PCR in combination with 16S-universal bacterial primers, namely 16S-27F and 16S-1391R. Primers for *Drosophila* RpL32 were used as a positive control. To analyse adult flies and eclosing rates after antibiotics treatment larvae were incubated for another 144 h. Wings, pupal cases and pupae of these specimens were mounted afterwards. Pictures were taken with a Leica MZ 9_5_ equipped with a LumixG2.

## Supplementary Material

Supplementary Material

## References

[b1] Bangi E. Pitsouli C. Rahme L. G. Cagan R. Apidianakis Y. (2012). Immune response to bacteria induces dissemination of Ras-activated Drosophila hindgut cells. EMBO Rep. 13, 569–576 10.1038/embor.2012.44 22498775 PMC3367237

[b2] Berry D. L. Baehrecke E. H. (2007). Growth arrest and autophagy are required for salivary gland cell degradation in Drosophila. Cell 131, 1137–1148 10.1016/j.cell.2007.10.048 18083103 PMC2180345

[b3] Bidla G. Dushay M. S. Theopold U. (2007). Crystal cell rupture after injury in Drosophila requires the JNK pathway, small GTPases and the TNF homolog Eiger. J. Cell Sci. 120, 1209–1215 10.1242/jcs.03420 17356067

[b4] Bidla G. Hauling T. Dushay M. S. Theopold U. (2009). Activation of insect phenoloxidase after injury: endogenous versus foreign elicitors. J. Innate Immun. 1, 301–308 10.1159/000168009 20375588

[b5] Brumby A. M. Richardson H. E. (2003). scribble mutants cooperate with oncogenic Ras or Notch to cause neoplastic overgrowth in Drosophila. EMBO J. 22, 5769–5779 10.1093/emboj/cdg548 14592975 PMC275405

[b6] Cordero J. B. Macagno J. P. Stefanatos R. K. Strathdee K. E. Cagan R. L. Vidal M. (2010). Oncogenic Ras diverts a host TNF tumor suppressor activity into tumor promoter. Dev. Cell 18, 999–1011 10.1016/j.devcel.2010.05.014 20627081 PMC3175220

[b7] Davis M. M. Engström Y. (2012). Immune response in the barrier epithelia: lessons from the fruit fly Drosophila melanogaster. J. Innate Immun. 4, 273–283 10.1159/000332947 22237424 PMC6741545

[b8] De Gregorio E. Spellman P. T. Tzou P. Rubin G. M. Lemaitre B. (2002). The Toll and Imd pathways are the major regulators of the immune response in Drosophila. EMBO J. 21, 2568–2579 10.1093/emboj/21.11.2568 12032070 PMC126042

[b9] Dvorak H. F. (1986). Tumors: wounds that do not heal. Similarities between tumor stroma generation and wound healing. N. Engl. J. Med. 315, 1650–1659 10.1056/NEJM198612253152606 3537791

[b10] Eleftherianos I. Revenis C. (2011). Role and importance of phenoloxidase in insect hemostasis. J. Innate Immun. 3, 28–33 10.1159/000321931 21051882

[b11] Feng Y. Santoriello C. Mione M. Hurlstone A. Martin P. (2010). Live imaging of innate immune cell sensing of transformed cells in zebrafish larvae: parallels between tumor initiation and wound inflammation. PLoS Biol. 8, e1000562 10.1371/journal.pbio.1000562 21179501 PMC3001901

[b12] Frey B. Gaipl U. S. (2011). The immune functions of phosphatidylserine in membranes of dying cells and microvesicles. Semin. Immunopathol. 33, 497–516. 20941495 10.1007/s00281-010-0228-6

[b13] Gateff E. (1978). Malignant neoplasms of genetic origin in Drosophila melanogaster. Science 200, 1448–1459 10.1126/science.96525 96525

[b14] Gong W. J. Golic K. G. (2006). Loss of Hsp70 in Drosophila is pleiotropic, with effects on thermotolerance, recovery from heat shock and neurodegeneration. Genetics 172, 275–286 10.1534/genetics.105.048793 16204210 PMC1456155

[b15] Gonzalez C. (2013). Drosophila melanogaster: a model and a tool to investigate malignancy and identify new therapeutics. Nat. Rev. Cancer 13, 172–183 10.1038/nrc3461 23388617

[b16] Govind S. (1996). Rel signalling pathway and the melanotic tumour phenotype of Drosophila. Biochem. Soc. Trans. 24, 39–44. 8674707 10.1042/bst0240039

[b17] Graveley B. R. Brooks A. N. Carlson J. W. Duff M. O. Landolin J. M. Yang L. Artieri C. G. van Baren M. J. Boley N. Booth B. W. (2011). The developmental transcriptome of Drosophila melanogaster. Nature 471, 473–479 10.1038/nature09715 21179090 PMC3075879

[b18] Halme A. Cheng M. Hariharan I. K. (2010). Retinoids regulate a developmental checkpoint for tissue regeneration in Drosophila. Curr. Biol. 20, 458–463 10.1016/j.cub.2010.01.038 20189388 PMC2847081

[b19] Hanahan D. Weinberg R. A. (2011). Hallmarks of cancer: the next generation. Cell 144, 646–674 10.1016/j.cell.2011.02.013 21376230

[b20] Huang D. W. Sherman B. T. Zheng X. Yang J. Imamichi T. Stephens R. Lempicki R. A. (2009). Extracting biological meaning from large gene lists with DAVID. Curr. Protoc. Bioinformatics 27, 13.11.1–13.11.13 10.1002/0471250953.bi1311s27 19728287

[b21] Igaki T. Pastor-Pareja J. C. Aonuma H. Miura M. Xu T. (2009). Intrinsic tumor suppression and epithelial maintenance by endocytic activation of Eiger/TNF signaling in Drosophila. Dev. Cell 16, 458–465 10.1016/j.devcel.2009.01.002 19289090 PMC2729686

[b22] Irving P. Ubeda J. M. Doucet D. Troxler L. Lagueux M. Zachary D. Hoffmann J. A. Hetru C. Meister M. (2005). New insights into Drosophila larval haemocyte functions through genome-wide analysis. Cell. Microbiol. 7, 335–350. 15679837 10.1111/j.1462-5822.2004.00462.x

[b23] Karim F. D. Rubin G. M. (1998). Ectopic expression of activated Ras1 induces hyperplastic growth and increased cell death in Drosophila imaginal tissues. Development 125, 1–9. 9389658 10.1242/dev.125.1.1

[b24] Karpac J. Younger A. Jasper H. (2011). Dynamic coordination of innate immune signaling and insulin signaling regulates systemic responses to localized DNA damage. Dev. Cell 20, 841–854 10.1016/j.devcel.2011.05.011 21664581 PMC3151532

[b25] Kounatidis I. Ligoxygakis P. (2012). Drosophila as a model system to unravel the layers of innate immunity to infection. Open Biol 2, 120075 10.1098/rsob.120075 22724070 PMC3376734

[b26] Kurucz E. Zettervall C. J. Sinka R. Vilmos P. Pivarcsi A. Ekengren S. Hegedüs Z. Ando I. Hultmark D. (2003). Hemese, a hemocyte-specific transmembrane protein, affects the cellular immune response in Drosophila. Proc. Natl. Acad. Sci. USA 100, 2622–2627 10.1073/pnas.0436940100 12598653 PMC151390

[b27] Kurucz E. Váczi B. Márkus R. Laurinyecz B. Vilmos P. Zsámboki J. Csorba K. Gateff E. Hultmark D. Andó I. (2007). Definition of Drosophila hemocyte subsets by cell-type specific antigens. Acta Biol. Hung. 58, Suppl., 95-111 10.1556/ABiol.58.2007.Suppl.8 18297797

[b28] Lee M. J. Mondal A. Small C. Paddibhatla I. Kawaguchi A. Govind S. (2011). A database for the analysis of immunity genes in Drosophila: PADMA database. Fly (Austin) 5, 155–161 10.4161/fly.5.2.14674 21273816 PMC3360108

[b29] Lemaitre B. Hoffmann J. (2007). The host defense of Drosophila melanogaster. Annu. Rev. Immunol. 25, 697–743 10.1146/annurev.immunol.25.022106.141615 17201680

[b30] Lemaitre B. Meister M. Govind S. Georgel P. Steward R. Reichhart J.-M. Hoffmann J. A. (1995). Functional analysis and regulation of nuclear import of dorsal during the immune response in Drosophila. EMBO J. 14, 536–545. 7859742 10.1002/j.1460-2075.1995.tb07029.x PMC398111

[b31] Márkus R. Laurinyecz B. Kurucz E. Honti V. Bajusz I. Sipos B. Somogyi K. Kronhamn J. Hultmark D. Andó I. (2009). Sessile hemocytes as a hematopoietic compartment in Drosophila melanogaster. Proc. Natl. Acad. Sci. USA 106, 4805–4809 10.1073/pnas.0801766106 19261847 PMC2660760

[b32] Miles W. O. Dyson N. J. Walker J. A. (2011). Modeling tumor invasion and metastasis in Drosophila. Dis. Model. Mech. 4, 753–761 10.1242/dmm.006908 21979943 PMC3209645

[b33] Minakhina S. Steward R. (2006). Melanotic mutants in Drosophila: pathways and phenotypes. Genetics 174, 253–263 10.1534/genetics.106.061978 16816412 PMC1569781

[b34] Nichols Z. Vogt R. G. (2008). The SNMP/CD36 gene family in Diptera, Hymenoptera and Coleoptera: Drosophila melanogaster, D. pseudoobscura, Anopheles gambiae, Aedes aegypti, Apis mellifera, and Tribolium castaneum. Insect Biochem. Mol. Biol. 38, 398–415 10.1016/j.ibmb.2007.11.003 18342246

[b35] Pagliarini R. A. Xu T. (2003). A genetic screen in Drosophila for metastatic behavior. Science 302, 1227–1231 10.1126/science.1088474 14551319

[b36] Pallavi S. K. Ho D. M. Hicks C. Miele L. Artavanis-Tsakonas S. (2012). Notch and Mef2 synergize to promote proliferation and metastasis through JNK signal activation in Drosophila. EMBO J. 31, 2895–2907 10.1038/emboj.2012.129 22580825 PMC3395089

[b37] Pastor-Pareja J. C. Grawe F. Martín-Blanco E. García-Bellido A. (2004). Invasive cell behavior during Drosophila imaginal disc eversion is mediated by the JNK signaling cascade. Dev. Cell 7, 387–399 10.1016/j.devcel.2004.07.022 15363413

[b38] Pastor-Pareja J. C. Wu M. Xu T. (2008). An innate immune response of blood cells to tumors and tissue damage in Drosophila. Dis. Model. Mech. **1**, 144-154, discussion 153 10.1242/dmm.000950 PMC2562178 19048077

[b39] Quiñones-Coello A. T. Petrella L. N. Ayers K. Melillo A. Mazzalupo S. Hudson A. M. Wang S. Castiblanco C. Buszczak M. Hoskins R. A. (2007). Exploring strategies for protein trapping in Drosophila. Genetics 175, 1089–1104 10.1534/genetics.106.065995 17179094 PMC1840052

[b40] Rajan A. Perrimon N. (2013). Of flies and men: insights on organismal metabolism from fruit flies. BMC Biol. 11, 38. 23587196 10.1186/1741-7007-11-38 PMC3626883

[b41] Reddy K. V. Yedery R. D. Aranha C. (2004). Antimicrobial peptides: premises and promises. Int. J. Antimicrob. Agents 24, 536–547 10.1016/j.ijantimicag.2004.09.005 15555874

[b42] Rera M. Clark R. I. Walker D. W. (2012). Intestinal barrier dysfunction links metabolic and inflammatory markers of aging to death in Drosophila. Proc. Natl. Acad. Sci. USA 109, 21528–21533 10.1073/pnas.1215849110 23236133 PMC3535647

[b43] Ryu J. H. Kim S. H. Lee H. Y. Bai J. Y. Nam Y. D. Bae J. W. Lee D. G. Shin S. C. Ha E. M. Lee W. J. (2008). Innate immune homeostasis by the homeobox gene caudal and commensal-gut mutualism in Drosophila. Science 319, 777–782 10.1126/science.1149357 18218863

[b44] Scherfer C. Qazi M. R. Takahashi K. Ueda R. Dushay M. S. Theopold U. Lemaitre B. (2006). The Toll immune-regulated Drosophila protein Fondue is involved in hemolymph clotting and puparium formation. Dev. Biol. 295, 156–163 10.1016/j.ydbio.2006.03.019 16690050

[b45] Schlenke T. A. Morales J. Govind S. Clark A. G. (2007). Contrasting infection strategies in generalist and specialist wasp parasitoids of Drosophila melanogaster. PLoS Pathog. 3, e158 10.1371/journal.ppat.0030158 PMC2042021 17967061

[b46] Schreiber R. D. Old L. J. Smyth M. J. (2011). Cancer immunoediting: integrating immunity's roles in cancer suppression and promotion. Science 331, 1565–1570 10.1126/science.1203486 21436444

[b47] Sorrentino R. P. (2010). Large standard deviations and logarithmic-normality: the truth about hemocyte counts in Drosophila. Fly (Austin) 4, 327–332 10.4161/fly.4.4.13260 20855971 PMC3174483

[b48] Srivastava A. Pastor-Pareja J. C. Igaki T. Pagliarini R. Xu T. (2007). Basement membrane remodeling is essential for Drosophila disc eversion and tumor invasion. Proc. Natl. Acad. Sci. USA 104, 2721–2726 10.1073/pnas.0611666104 17301221 PMC1815248

[b49] Theopold U. Krautz R. Dushay M. S. (2014). The Drosophila clotting system and its messages for mammals. Dev. Comp. Immunol. 42, 42–46 10.1016/j.dci.2013.03.014 23545286

[b50] Trapnell C. Pachter L. Salzberg S. L. (2009). TopHat: discovering splice junctions with RNA-Seq. Bioinformatics 25, 1105–1111 10.1093/bioinformatics/btp120 19289445 PMC2672628

[b51] Trapnell C. Roberts A. Goff L. Pertea G. Kim D. Kelley D. R. Pimentel H. Salzberg S. L. Rinn J. L. Pachter L. (2012). Differential gene and transcript expression analysis of RNA-seq experiments with TopHat and Cufflinks. Nat. Protoc. 7, 562–578 10.1038/nprot.2012.016 22383036 PMC3334321

[b52] Tung T. T. Nagaosa K. Fujita Y. Kita A. Mori H. Okada R. Nonaka S. Nakanishi Y. (2013). Phosphatidylserine recognition and induction of apoptotic cell clearance by Drosophila engulfment receptor Draper. J. Biochem. 153, 483–491 10.1093/jb/mvt014 23420848

[b53] Uhlirova M. Bohmann D. (2006). JNK- and Fos-regulated Mmp1 expression cooperates with Ras to induce invasive tumors in Drosophila. EMBO J. 25, 5294–5304 10.1038/sj.emboj.7601401 17082773 PMC1636619

[b54] Uttenweiler-Joseph S. Moniatte M. Lagueux M. Van Dorsselaer A. Hoffmann J. A. Bulet P. (1998). Differential display of peptides induced during the immune response of Drosophila: a matrix-assisted laser desorption ionization time-of-flight mass spectrometry study. Proc. Natl. Acad. Sci. USA 95, 11342–11347 10.1073/pnas.95.19.11342 9736738 PMC21644

[b55] Vodovar N. Vinals M. Liehl P. Basset A. Degrouard J. Spellman P. Boccard F. Lemaitre B. (2005). Drosophila host defense after oral infection by an entomopathogenic Pseudomonas species. Proc. Natl. Acad. Sci. USA 102, 11414–11419 10.1073/pnas.0502240102 16061818 PMC1183552

[b55a] Wang T. Jiao Y. Montell C. (2007). Dissection of the pathway required for generation of vitamin A and for Drosophila phototransduction. J. Cell Biol. 177, 305–316 10.1083/jcb.200610081 17452532 PMC2064138

[b56] Watson K. L. Johnson T. K. Denell R. E. (1991). Lethal(1) aberrant immune response mutations leading to melanotic tumor formation in Drosophila melanogaster. Dev. Genet. 12, 173–187 10.1002/dvg.1020120302 1907895

[b57] Watson K. L. Konrad K. D. Woods D. F. Bryant P. J. (1992). Drosophila homolog of the human S6 ribosomal protein is required for tumor suppression in the hematopoietic system. Proc. Natl. Acad. Sci. USA 89, 11302–11306 10.1073/pnas.89.23.11302 1454811 PMC50538

[b58] Watson K. L. Justice R. W. Bryant P. J. (1994). Drosophila in cancer research: the first fifty tumor suppressor genes. J. Cell Sci. Suppl. 18, 19–33 10.1242/jcs.1994.Supplement_18.4 7883789

[b59] Wertheim B. Kraaijeveld A. R. Schuster E. Blanc E. Hopkins M. Pletcher S. D. Strand M. R. Partridge L. Godfray H. C. (2005). Genome-wide gene expression in response to parasitoid attack in Drosophila. Genome Biol. 6, R94. 16277749 10.1186/gb-2005-6-11-r94 PMC1297650

[b60] Williams M. J. (2007). Drosophila hemopoiesis and cellular immunity. J. Immunol. 178, 4711–4716. 17404248 10.4049/jimmunol.178.8.4711

[b61] Zwaal R. F. Comfurius P. Bevers E. M. (2005). Surface exposure of phosphatidylserine in pathological cells. Cell. Mol. Life Sci. 62, 971–988. 15761668 10.1007/s00018-005-4527-3 PMC11924510

