# Supplementary material for: A Drosophila immune response against Ras-induced overgrowth
Source: Biol Open. 2014 Mar 21;3(4):250–60. doi: 10.1242/bio.20146494 (PMC3988794; doi:10.1242/bio.20146494)
Supplement: Supplementary Material [file supp_3_4_250__index.html]

A Drosophila immune response against Ras-induced overgrowth — Supplementary Material 

# A *Drosophila* immune response against Ras-induced overgrowth

## bio.20146494 Supplementary Material

**Files in this Data Supplement:**

- Supplementary Material - Thomas Hauling et al. doi: 10.1242/bio.20146494
- Table S1 - Complete list of Ras-induced genes and comparison with other infection models. The complete list of differentially regulated genes is shown, genes that differ at q<0.05 are highlighted in green, genes that differ at p<0.05 are indicated in red letters. The list of regulated genes is also compared to those after infection with bacteria and after wasp infestation (see supplementary material Fig. S4 for a Venn diagram of this comparison).
- Table S2 - Genes associated with immunity are up-regulated in RasV12-expressing larvae. A gene ontology cluster analysis of all genes showing significant changes (up- and down-regulated) in the differential gene expression analysis was performed using DAVID (Huang et al., 2009).
- Table S3 - Melanization of apoptotic tissues. The induction of apoptotic inducers was carried out as described in Materials and Methods for UAS-RasV12. The observed phenotypes were as follows: (−): no melanization; (o): small melanotic masses; (+): large melanotic masses (additional observations are indicated in brackets).
