# Supplementary material for: A Drosophila immune response against Ras-induced overgrowth
Source: Biol Open. 2014 Mar 21;3(4):250–60. doi: 10.1242/bio.20146494 (PMC3988794; doi:10.1242/bio.20146494)
Supplement: Supplementary Material [file supp_bio.20146494_bio.20146494-s1.pdf]

Biology Open (2014) 000, 1–11 doi:10.1242/bio.20146494

**Supplementary Material**

**Thomas Hauling et al. doi: 10.1242/bio.20146494**

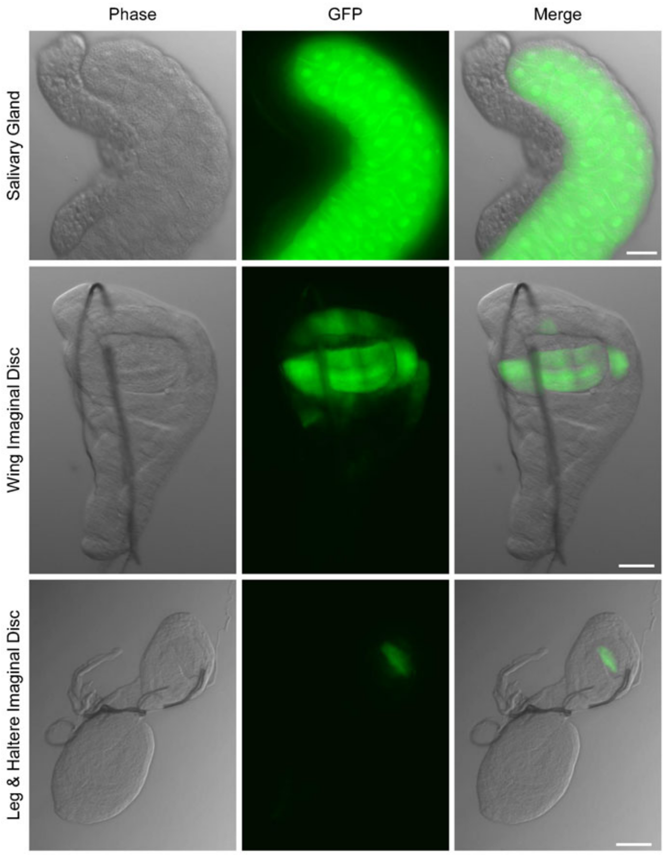


**Fig. S1. Expression pattern of the Bx-Gal4 driver revealed in crosses with the UAS-GFP transgene in tissues that score positive.** Phase contrast (left), fluorescence (middle) and merged exposures are shown. Scale bars: 50 µm.

Biology Open (2014) 000, 1–11 doi:10.1242/bio.20146494

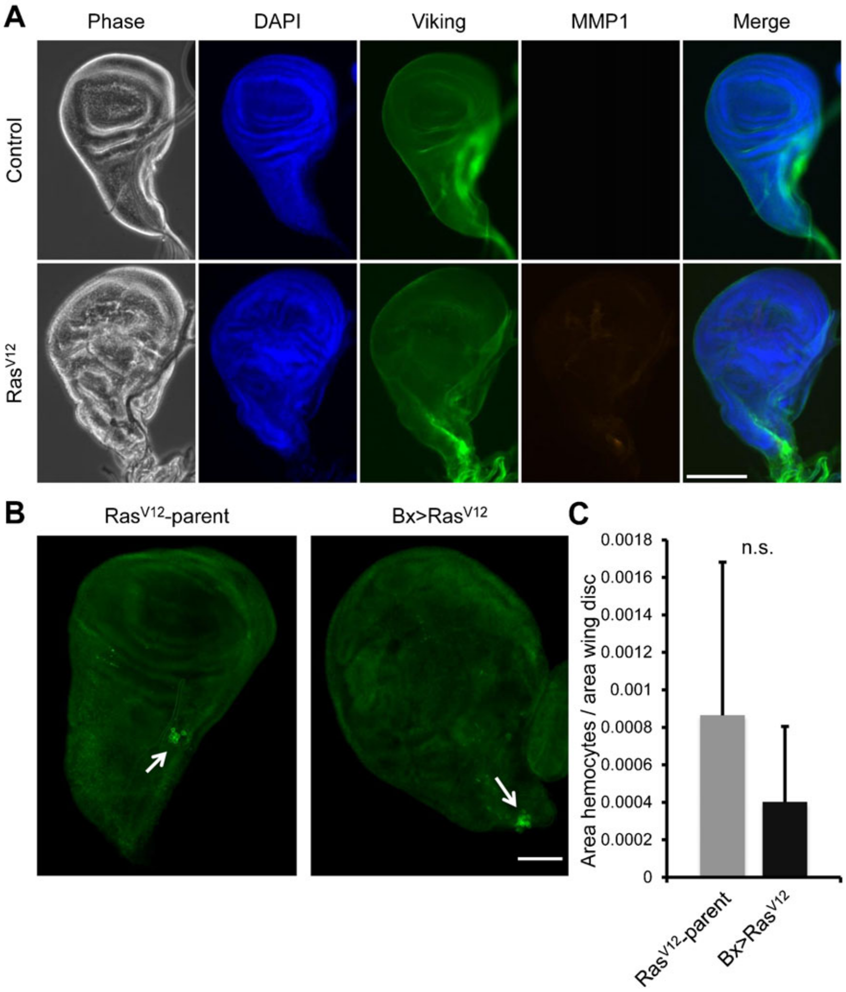


**Fig. S2. Imaginal discs from Ras$^{V12}$-expressing larvae produce little MMP1 and hemocyte occupation on control and Ras$^{V12}$ wing imaginal discs does not differ significantly.** Wing imaginal discs (A) from control crosses (Bx;+/+: Ctrl) and Ras-expressing larvae (Bx>Ras, labeled Ras$^{V12}$ in the figure) are shown in phase contrast, after nuclear staining (DAPI), in the green channel showing expression of a Viking–GFP fusion protein (Vkg::GFP) and after staining with an MMP1-specific antibody. (B) Control (UAS-Ras$^{V12}$ parent line, left) wing disc with hemocytes (red arrow) and Ras$^{V12}$ wing disc with hemocytes (Bx>Ras$^{V12}$, right part). (C) The ratio between areas occupied by hemocytes and total average disc area does not differ significantly between the two genotypes (n.s., $p=0.531630$, means ± SD). The ratio between areas was determined using ImageJ. Scale bars: 100 µm.

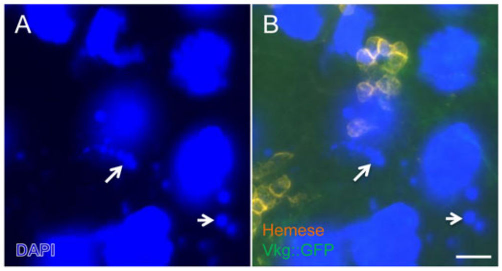


**Fig. S3. DNA fragmentation in Ras-expressing salivary glands.** A larger magnification of glands such as shown in Fig. 2A is shown after staining with DAPI (blue) (A), and a hemocyte-specific antibody (red) (B). Two nuclear fragments are indicated by arrows (note that the nuclear fragments do not co-localize with the hemocyte signal). Scale bar: 20 µm.

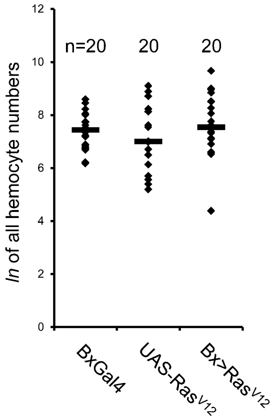


**Fig. S4. Total hemocyte counts in Ras-expressing and control larvae.** The *ln* of all hemocyte numbers shows a lack of hemocyte proliferation. The dots show individual values. *n*: number of individuals analyzed. No difference between samples was detected ($p=0.187$).

Biology Open

Biology Open (2014) 000, 1–11 doi:10.1242/bio.20146494

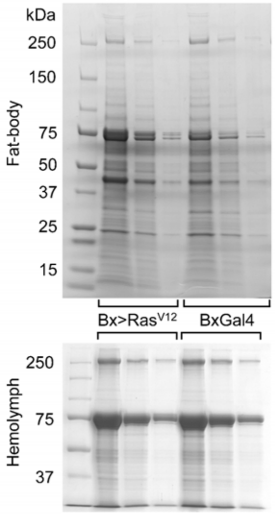


**Fig. S5. Similar protein patterns in fat body and hemolymph from control larvae and larvae, which express Ras$^{V12}$ in salivary glands.** Proteins from fat body lysates (upper) and hemolymph proteins (lower) were compared between the indicated crosses on a gradient (fat body) and a 6% polyacrylamide gel (hemolymph). Molecular masses are indicated. (The equivalent of 20% of the fat body (upper) from one larva and 1 µl hemolymph (lower) was loaded and two additional threefold dilutions of the same sample).

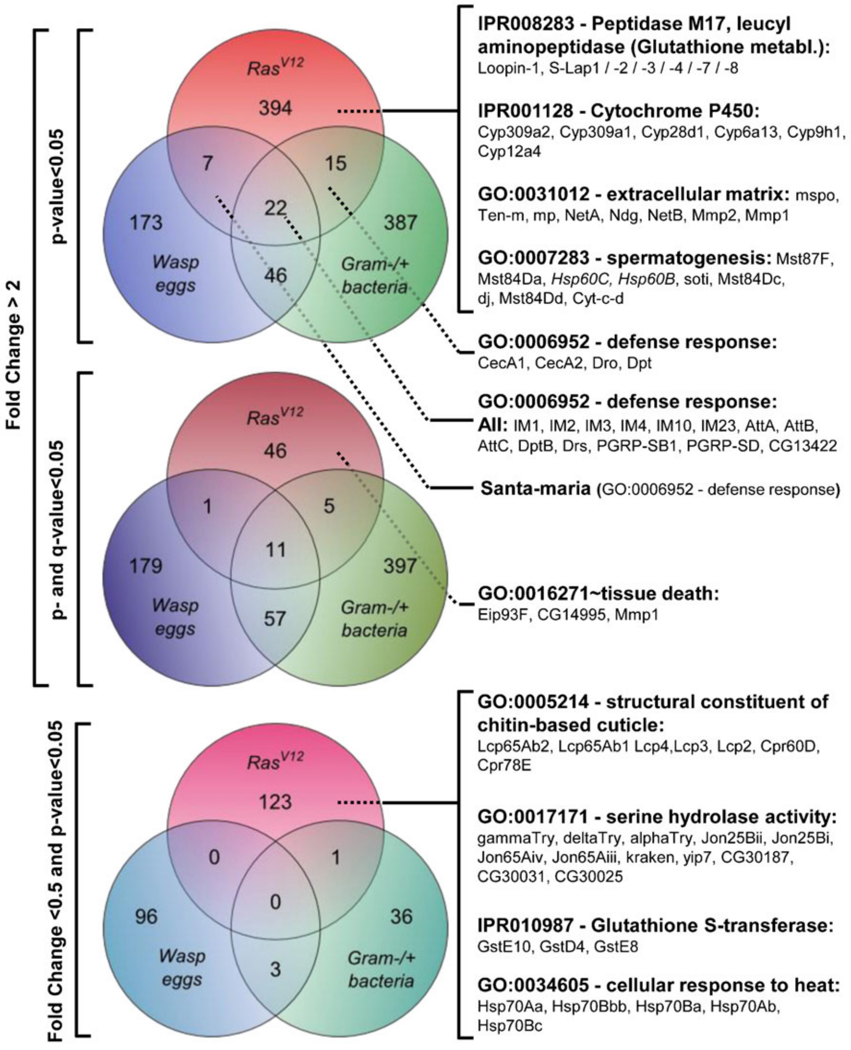


**Fig. S6. Venn diagram showing the overlap of differentially expressed genes after expression of Ras and after bacterial infection and wasp infestation.** Comparison of bacterially- (Irving et al., 2005; Vodovar et al., 2005), wasp- (Lee et al., 2011; Schlenke et al., 2007; Wertheim et al., 2005) and Ras$^{V12}$-induced genes in a Venn diagram shows a core component of shared immune genes (altogether 17 genes in the central figure) as well as additional genes shared between Ras-expressing and bacterially- or wasp-infected larvae. The most significant GO classifications for selected categories are also indicated (see supplementary material Table S2 for a complete survey).

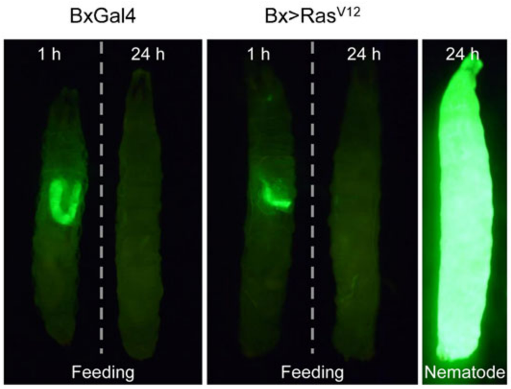


**Fig. S7. Lack of septicemia in Ras$^{V12}$-expressing larvae.** Control larvae (Bx-GAL4 crossed to w$^{1118}$) and Ras$^{V12}$-expressing larvae (Bx-GAL4>UAS-Ras$^{V12}$) were fed with entomopathogenic *Photorhabdus luminescence*, which were added to the food or by introducing the same bacteria via nematode infection as a positive control (24 h).

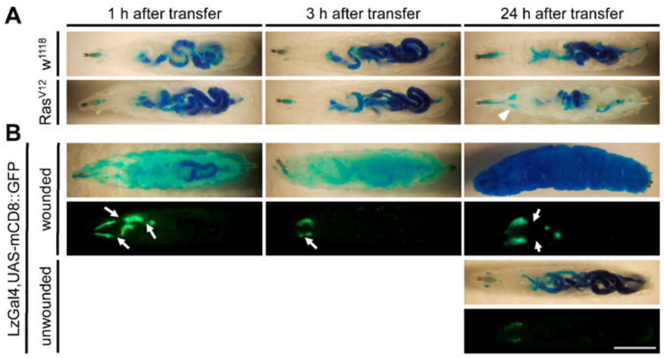


**Fig. S8. Ras-expressing glands are impermeable to small compounds.** (A) Larvae expressing Ras and larvae from control crosses were transferred to medium containing dye and subsequently scanned for presence of the dye in the hemolymph after the indicated periods. Arrow tips point to dye inside the salivary gland lumen. (B) Larvae with strong expression of GFP in the salivary glands (using the lozenge driver) were injured by aseptic in situ wounding (pinch wounds). Arrows point to constriction or disruption sites along the salivary gland, introduced. Scale bar: 1 mm.

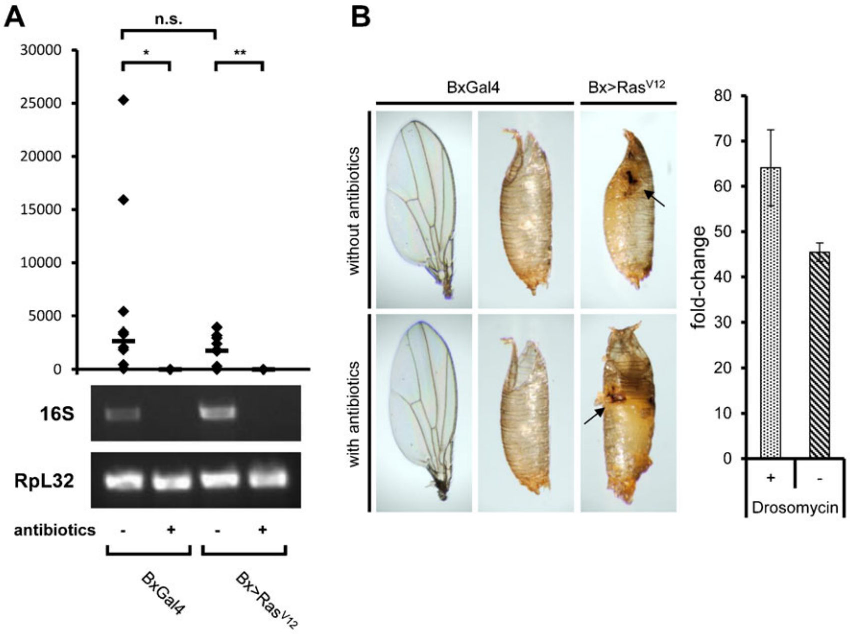


**Fig. S9. Drosomcyin is expressed in Ras-expressing larvae in the absence of bacteria.** (A) Ras-expressing and control larvae were grown with and without antibiotics. Depletion of bacteria was checked by PCR using 16S universal primers and primers specific for *Drosophila* ribosomal protein 32 as control. (B) Left: Ras-expressing shows melanotic spots (arrows, compare with Fig. 1) even in the absence of bacteria, while control larvae display normal wing phenotypes and hatching rate. The pupal cases show no signs of melanization. (B) Right: Ras-expressing larvae were kept under conditions with (+) and without antibiotics (−) and the induction of Drosomycin monitored, indicating that *Drs* induction is not dependent on the presence of bacteria. Note that *Drs* expression is not significantly different after antibiotics treatment (means ± SD, p=0.379).
