# Supplementary material for: A Drosophila immune response against Ras-induced overgrowth
Source: Biol Open. 2014 Mar 21;3(4):250–60. doi: 10.1242/bio.20146494 (PMC3988794; doi:10.1242/bio.20146494)
Supplement: Supplementary Material [file supp_bio.20146494_Table_S3.docx]

**Table S3. Melanization of apoptotic tissues**

|  | Expression profile of GAL 4 driver (additional information in brackets) | Melanization (additional observations in brackets) |
| --- | --- | --- |
| UAS-grim | imaginal discs, brain (c701b-GAL4) | − (lethal) |
|  | eye disc (GmR-GAL4) | − (reduced eyes in adults) |
|  | wing disc and salivary glands (BxMS1096-GAL4) | − (extended larval period, reduced wings in adults) |
|  | salivary glands (Sgs3-GAL4) | − |
|  | late 3rd instar fat body (Lsp2-GAL4) | − (larvae more transparent) |
|  | fat body and salivary glands (5253 GAL4) | − (lethal in larval stage, larvae are smaller than wt) |
|  | hemocytes (he-GAL4: tub-GAL80ts) | + (melanotic bodies in hemolymph) |
|  | hemocytes (he-GAL4) | o (melanotic bodies in hemolymph) |
|  | ubiquitously in all tissues (Heat shock GAL4 (heat shock at 37°C for 1 h, then transfer to 25°C) | + (lethal, melanization variable, throughout the animal) |
| UAS-hid | imaginal discs, brain (c701b-GAL4) | lethal |
|  | late 3rd instar fat body (Lsp2-GAL4) | − |
|  | fat body and salivary glands (5253 GAL4) | − (lethal in larval stage) |
|  | eye disc (GmR-GAL4) | − (lethal in pupal stage) |
| UAS-reaper | late 3rd instar fat body (Lsp2-GAL4) | − |
|  | eye disc (GmR-GAL4) | − (lethal in pupal stage) |

The induction of apoptotic inducers was carried out as described in Materials and Methods for UAS-Ras^V12^. The observed phenotypes were as follows: (−): no melanization; (o): small melanotic masses; (+): large melanotic masses (additional observations are indicated in brackets).
